# C1q/TNF‐related protein 9 inhibits the cholesterol‐induced Vascular smooth muscle cell phenotype switch and cell dysfunction by activating AMP‐dependent kinase

**DOI:** 10.1111/jcmm.13196

**Published:** 2017-05-19

**Authors:** Qi Liu, Hui Zhang, Jiale Lin, Ruoxi Zhang, Shuyuan Chen, Wei Liu, Meng Sun, Wenjuan Du, Jingbo Hou, Bo Yu

**Affiliations:** ^1^ The Key Laboratory of Myocardial Ischemia Organization Chinese Ministry of Education Harbin China; ^2^ Division Department of Cardiology Organization The Second Affiliated Hospital of Harbin Medical University Harbin China

**Keywords:** C1q/TNF‐related protein 9, Vascular smooth muscle cell, macrophage‐like cells, cholesterol, AMP‐dependent kinase

## Abstract

Vascular smooth muscle cells (VSMCs) switch to macrophage‐like cells after cholesterol loading, and this change may play an important role in the progression of atherosclerosis. C1q/TNF‐related protein 9 (CTRP9) is a recently discovered adipokine that has been shown to have beneficial effects on glucose metabolism and vascular function, particularly in regard to cardiovascular disease. The question of whether CTRP9 can protect VSMCs from cholesterol damage has not been addressed. In this study, the impact of CTRP9 on cholesterol‐damaged VSMCs was observed. Our data show that in cholesterol‐treated VSMCs, CTRP9 significantly reversed the cholesterol‐induced increases in pro‐inflammatory factor secretion, monocyte adhesion, cholesterol uptake and expression of the macrophage marker CD68. Meanwhile, CTRP9 prevented the cholesterol‐induced activation of the TLR4–MyD88–p65 pathway and upregulated the expression of proteins important for cholesterol efflux. Mechanistically, as siRNA‐induced selective gene ablation of AMPKα1 abolished these effects of CTRP9, we concluded that CTRP9 achieves these protective effects in VSMCs through the AMP‐dependent kinase (AMPK) pathway.

## Introduction

Atherosclerosis is a leading cause of morbidity and mortality around the world 1, 2. Atherosclerotic plaque lumen‐narrowing and rupture‐induced thrombosis or obstruction of the coronary artery are the biggest causes of the sudden and unpredictable onset of acute coronary syndrome 3, 4.

One of the features of an unstable plaque is the presence of a large necrotic core containing cells filled with lipid (foam cells) 4. It is widely accepted that the majority of foam cells are derived from macrophages that originate from circulating monocytes; however, recent research has provided a different vision for the origin of some macrophages; VSMCs that become loaded with cholesterol have been shown to lose VSMC markers such as smooth muscle α‐actin (ACTA2) and assume the appearance of macrophages expressing macrophage‐specific markers such as CD68 5, 6. In fact, in human atherosclerotic plaques, 30–40 percent of cells positive for the macrophage marker CD68 are VSMC‐derived 5. The athero‐protective layer, a fibrous cap, consists of ACTA2‐positive cells that surround the atherosclerotic plaque. Accordingly, the role of VSMCs in the atherogenic process has hitherto been underestimated 7, 8. Therefore, changes in VSMC phenotype can play a critical role in lesion development, plaque composition and stability. Furthermore, therapeutics aimed at inhibiting cholesterol injure in VSMCs may be a feasible means of treating atherosclerosis.

CTRP9, an adipocytokine that has the highest amino acid identity to adiponectin, is expressed in adipose tissue 9, 10 and functions through activating AMP‐dependent kinase (AMPK). It is well known that CTRP9 has multiple functions, including regulating glucose and lipid metabolism 9, 11, 12, 13, protecting against ischaemia–reperfusion injury 14, 15, 16, 17, 18, acting as an anti‐inflammatory agent and preventing oxidative damage 19, 20, 21. In particular, CTRP9 has been reported to have beneficial effects in the cardiovascular system. In this regard, previous studies have shown that CTRP9 can enhance plaque stability by enhancing endothelium‐dependent vaso‐relaxation 22, attenuating VSMC proliferation, formation of the neointima 23, and reducing pro‐inflammatory cytokine expression 24, 25. Although CTRP9 is abundantly expressed in epicardial adipose tissue 26 and cardiac endothelial cells 27 in the cardiovascular system, it may also exert its effects on coronary arterial cells through paracrine and vasocrine means, similar to the action of other adipokines. Given the protective role CTRP9 plays in the cardiovascular system, it was of interest to understand whether CTRP9 could also play a protective role against the cholesterol‐induced changes in VSMCs that leads to a switch to a macrophage‐like phenotype. In this study, we found that in VSMCs, CTRP9 inhibits the cholesterol‐mediated switch to dysfunctional macrophage‐like cells, thereby protecting them from cholesterol damage.

## Materials and methods

### Materials

Human aortic VSMCs (#6110) and smooth muscle cell medium (SMCM, 1101) were purchased from ScienCell (San Diego, CA, USA). Recombinant human CTRP9 (6537‐TN‐050) and all ELISA‐based detection systems (IL‐6, VAL102, IL‐1β, VAL101 and MCP‐1, DCP00) were purchased from R&D Systems (Minneapolis, MN, USA). Cholesterol–methyl‐β‐cyclodextrin (C4951), paraformaldehyde (PFA), IGEPAL CA‐630, Oil Red O and Harris’ hematoxylin, bovine serum albumin (BSA) and the Cholesterol Quantitation Kit (MAK043) were obtained from Sigma‐Aldrich (St. Louis, MO, USA). Cell Counting Kit 8 (CCK8) was obtained from Dojindo (Kumamoto, Japan). Trizol and CELLTRACE Violet were obtained from Invitrogen (Carlsbad, CA, USA). DAPI, Transcriptor First Strand cDNA Synthesis Kit, FastStart Universal SYBR Green Master Mix and X‐treme siRNA Transfection Reagent were obtained from Roche (Penzberg, Germany). THP1 cells were obtained from FuDan IBS Cell Center (Shanghai, China). Both negative control siRNA (siRNA [NC]) and an siRNA targeting AMPK α1 (siRNA [α1]) (GCAGAAGTATGTAGAGCAA) were synthesized by RiboBio (Guangzhou, China). All of the reagents and antibodies (FITC mouse anti‐human CD68 (562117) and isotype control [555057]) used in for flow cytometry assay were obtained from BD Biosciences (Franklin Lakes, NJ, USA). Primary antibodies against ACTA (ab5694), CD68 (ab955), ICAM‐1 (ab53013), VCAM‐1 (ab134047), LOX‐1 (ab126538), LXR (ab176323), ABCA1 (ab18180), ABCG1 (ab52617), MyD88 (ab2068) and TLR4 (ab13556), and fluorescently labelled secondary antibodies (ab6270 and ab6785) were obtained from Abcam (Cambridge, MA, USA). Primary antibodies against phospho‐P65 (#3033), total‐P65 (#8242), phospho‐AMPK (#2535) and total AMPK (#2532) were obtained from Cell Signaling Technology (Danvers, MA, USA). The β‐actin antibody (sc‐47778) and peroxidase‐conjugated goat anti‐rabbit IgG or anti‐mouse IgG were obtained from ZSGB‐BIO (Beijing, China).

### Cell culture

VSMCs were cultured in SMCM containing 2% foetal bovine serum (FBS), 1% smooth muscle cell growth supplement (SMCGS), 100 units/ml penicillin and 100 μg/ml streptomycin, at 37°C in a 5% CO_2_ humidified atmosphere. In all experiments, VSMCs between passages 3 and 6 were used.

### VSMC synchronization

Following growth of VSMCs to 80% confluence, cells were synchronized by changing the culture media to FBS‐ and SMCGS‐free SMCM basic culture media, and culturing at 37°C in a 5% CO_2_ humidified atmosphere for 24 hrs.

### CTRP9 pre‐treatment of VSMCs

Recombinant human CTRP9 was produced in a mouse myeloma cell line and demonstrated a purity >95% (R&D Systems). CTRP9 was reconstituted at a concentration of 250 μg/ml in sterile PBS. After cell synchronization, VSMCs were incubated with fresh serum‐free medium containing CTRP9 at the indicated concentrations and cultured for further 30 min before cholesterol treatment.

### Cholesterol treatment of VSMCs

Cholesterol was delivered to VSMCs using a water‐soluble cholesterol analog called cholesterol–methyl‐β‐cyclodextrin. After cell synchronization, the cell medium was changed to the cholesterol‐containing serum‐free media and cultured for 72 hrs, after which the cells were harvested for analysis.

### Cell viability assay using CCK8

VSMCs were seeded into 96‐well plates at a density of 2000 cells/well and cultured with different concentrations of cholesterol or CTRP9 for 72 hrs. Afterwards, 10 μl of CCK8 solution was added to each well, and the cells incubated for 2 hrs. The colour intensity was measured at an absorbance of 450 nm (Tecan Infinite M200 microplate reader; LabX, Austria). All experiments were performed in triplicate and were repeated three times.

### Oil red O staining

VSMCs were washed with PBS and fixed in 4% PFA (w/v dissolved in PBS). Following three washes, the cells were then incubated in 60% isopropanol for 3 min. After complete drying of the cells, cells were stained with Oil Red O and Harris’ hematoxylin. Images were captured under a microscope at 200× magnification (DMI4000B; Leica, Wetzlar, Germany).

### qRT‐PCR

Total RNA was extracted using Trizol according to the manufacturer's instructions. First‐strand cDNA was generated from 4 μg RNA using the Transcriptor First Strand cDNA Synthesis Kit. qRT‐PCR experiments were performed using the FastStart Universal SYBR Green Master Mix and a quantitative fluorescence PCR system 28. Glyceraldehyde 3‐phosphate dehydrogenase (GAPDH) or β‐actin served as an internal control for quantification of mRNAs. Each sample was measured in triplicate. qPCR data were analysed, and the expression of mRNA normalized relative to GAPDH or β‐actin was determined using the 2^−ΔΔCt^ cycle threshold method 28. Table 1 presents all related gene sequences.

**Table 1:** Primers for qRT‐PCR

Name		Sequence
ACTA2	Forward	5′‐GGTGATGGTGGGAATGGG‐3′
	Reverse	5′‐GCAGGGGTGGGATGCTCTT‐3′
CD68	Forward	5′‐CCTCTCATCATCGGCCTGAT‐3′
	Reverse	5′‐TCCGGATGATGCAGAAAGC‐3′
LGALS3	Forward	5′‐TCGCATGCTGATAACAATTCTG‐3′
	Reverse	5′‐AAGCGTGGGTTAAAGTGGAA‐3′
AMPK‐α	Forward	5′‐TGATGTTGTAGTGACACCATTTAC‐3′
	Reverse	5′‐GAAGATGAGGGAAAGAATTAAGGG‐3′
GAPDH	Forward	5′‐GGAGCGAGATCCCTCCAAAAT‐3′
	Reverse	5′‐GGCTGTTGTCATACTTCTCATGG‐3′
β‐actin	Forward	5′‐TCATGAAGTGTGTGACGTGGACATC‐3′
	Reverse	5′‐CAGCAGGAGCAATGATCTTGATCT‐3′

ACTA2 smooth muscle α‐actin; LGALS3 galectin 3.

### Enzyme‐linked immunosorbent assay

To measure the release of cytokines from VSMCs, supernatants were collected, and the levels of IL‐6, IL‐1β and MCP‐1 were determined by ELISA according to the manufacturer's instructions. All assays were performed in triplicate.

### Cell adhesion

VSMCs were incubated with different concentrations of CTRP9, with or without cholesterol, and cultured for 72 hrs as appropriate. Treated VSMCs were washed three times with PBS, and CELLTRACE Violet‐labelled THP1 cells (1 × 10^6^ cells per well) were added to the VSMC cultures and incubated for 1 hr at 10 r.p.m. at 37°C. Cells were then washed twice with FBS to remove non‐attached cells. The VSMC layers with attached monocytes were fixed with 4% PFA, and adhered THP1 cells were measured using a fluorescence microscope at 50× magnification (DMI4000B).

### siRNA transfection

The siRNAs were transfected into VSMCs using the X‐treme siRNA Transfection Reagent according to the manufacturer's instructions. The transfection reagent was mixed with serum‐free media containing 50 μM of either siRNA (α1) or siRNA (NC), and incubated for 15 min. at room temperature before adding to the VSMCs and incubating for a further 6 hrs. Forty‐eight hours later, the VSMCs were exposed to CTRP9 (10 μg/ml) and cholesterol (5 μg/ml) for 72 hrs.

### Intracellular total cholesterol measurement

Intracellular total cholesterol was determined using a Cholesterol Quantitation Kit, according to the manufacturer's instructions. VSMCs (1 × 10^6^ cells) were extracted with 200 μl of chloroform:isopropanol:octylphenoxy poly (ethyleneoxy) ethanol (IGEPAL CA‐630) (7:11:0.1) in a micro‐homogenizer. After centrifugation, the lipids were air‐dried, vacuum‐dried, dissolved and mixed until homogenous. The mixture was used to determine total cholesterol (μg/μl) using a colorimetric assay. Three independent experiments were conducted.

### Flow cytometry

The phenotype of the macrophage‐like cells was analysed by measuring the surface expression of the macrophage‐specific marker CD68 using flow cytometry. After appropriate treatments, VSMCs were collected and stained with an anti‐CD68 antibody (5 μl in 50 μl Perm/Wash buffer) or its isotype control (1 μl in 50 μl Perm/Wash buffer) incubated at 4°C for 30 min. in the dark. The stained cells were then washed and analysed using the FACSCanto II system equipped with BD FACSDiva software (Becton‐Dickinson, San Jose, CA, USA) to determine the percentage of CD68‐positive cells.

### Immunofluorescence

To characterize the VSMC phenotype, cells were fixed and incubated at 4°C overnight with primary antibodies against ACTA2 (1:200) or CD68 (1:200). Cells were then washed and incubated with the appropriate fluorescently labelled secondary antibodies (1:200) at 30°C in dark. All antibodies were diluted in 5% BSA (w/v) (diluted in PBS). Cells were then washed, and the nuclei were counterstained with DAPI. Cells were examined under a fluorescence microscope (DMI4000B) at 200× magnification.

### Western blotting

Western blotting was used to assess the expression of proteins related to the NF‐kB pathway, AMPK phosphorylation, cholesterol uptake and efflux, and cell adhesion in VSMCs. Briefly, equal amounts of total cell lysates were blotted onto a PVDF membrane and incubated with primary antibodies overnight at 4°C, and membranes were washed and then incubated with peroxidase‐conjugated second antibody for 1 hr at 37°C. Immuno‐complexes were visualized on a Luminescent Imaging Workstation (Tanon, Shanghai, China; 6600) using Electro‐Chemi‐Luminescence (ECL) detection with BeyoECL Plus (Beyotime Institute of Biotechnology, Beijing, China). Protein levels were quantified using scanning densitometry (Image J, National Institutes of Health, Bethesda, Rockville, MD, USA). Three independent experiments were conducted.

### Statistical analysis

Data were expressed as mean ± S.D. One‐way anova followed by Student's *t*‐test was used for multiple comparisons. A value of *P* < 0.05 was considered statistically significant. Data were analysed using GraphPad Prism software (GraphPad Inc, San Diego, CA, USA).

## Results

### Cholesterol uptake by VSMCs converts them to a macrophage foam cell‐like state

**Figure 1:**
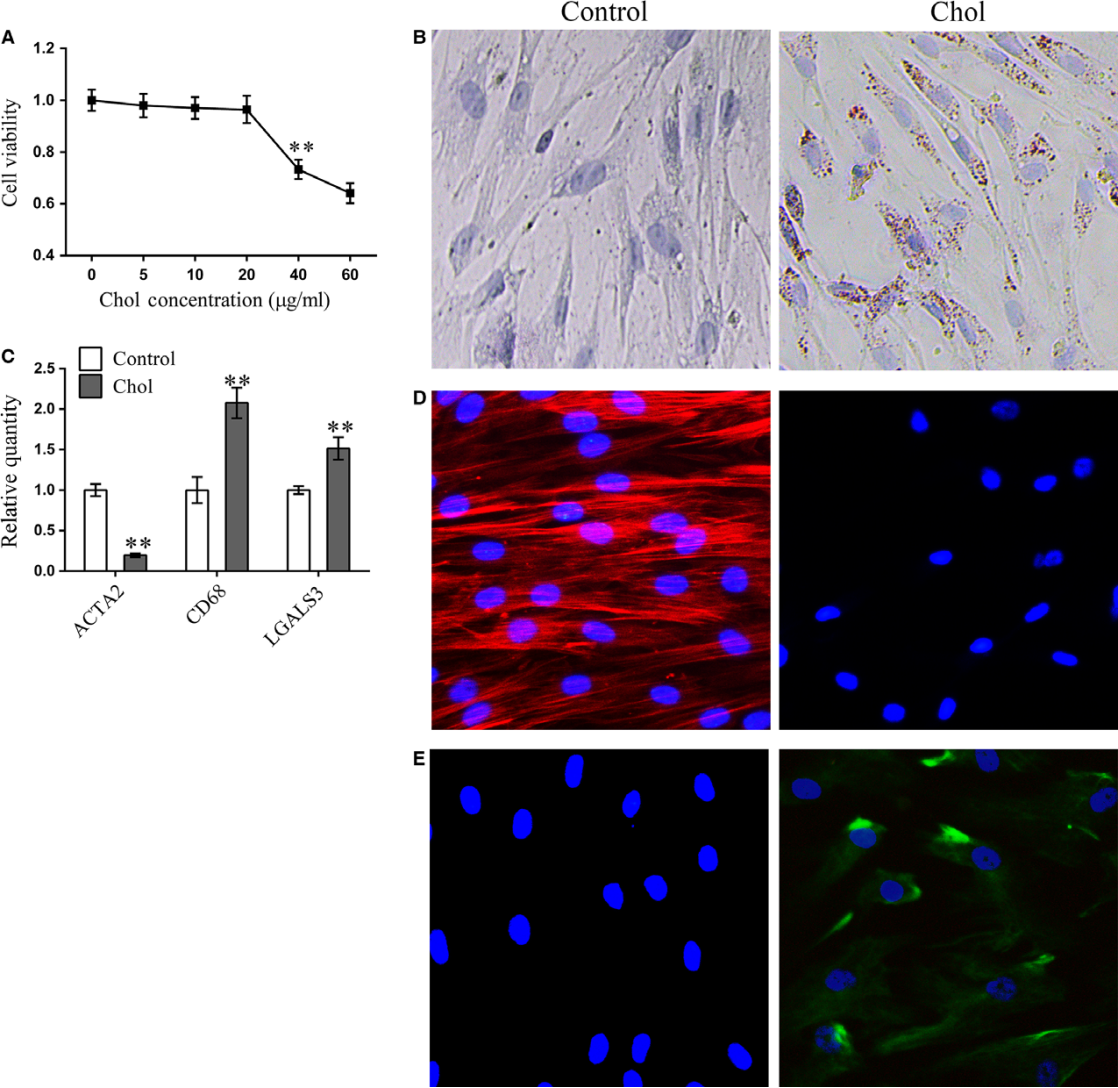
Cholesterol loading in VSMC leads to the accumulation of intracellular lipid droplets, the induction of macrophage‐related markers and a reduction in VSMC markers (**A**) Cell viability, measured using CCK‐8, in VSMC cells after treatment for 72 hrs with different concentrations of a cyclodextrin (CD)–cholesterol complex. (**B**) Representative images of Oil Red O and Harris’ hematoxylin‐stained VSMC cells. (**C**) mRNA expression of ACTA2, CD68 and LGALS3 measured by qRT‐PCR in VSMC cells treated with (5 μg/ml for 72 hrs) and without cholesterol. (**D**) Representative immunofluorescent images of ACTA2 expression in VSMC cells with (5 μg/ml for 72 hrs) and without cholesterol treatment. (**E**) Representative immunofluorescent images of CD68 expression in VSMC cells with (5 μg/ml for 72 hrs) and without cholesterol treatment. For CCK‐8 (**A**) and qRT‐PCR (**B**), data are shown as mean ± S.D. of triplicates and are representative of three independent experiments. **P < 0.01 *versus*. control group.

To examine the effects of cholesterol loading on the VSMC phenotype switch, we initially analysed multiple parameters including VSMC cell viability, lipid accumulation and expression of macrophage markers. The CCK8 assay was used to detect the effect of cholesterol on VSMC viability. Concentrations of cholesterol up to 20 μg/ml were not toxic to VSMCs, but concentrations above this had a clear toxic effect (Fig. 1A). Consistent with the results of a previous study 8, after incubation with 5 μg/ml cholesterol for 72 hrs, Oil‐Red‐O‐stainable lipid droplets appeared in VSMCs (Fig. 1B), demonstrating the formation of foam cells. To further characterize this VSMC phenotypic switch, qRT‐PCR was used to assess the levels of a smooth muscle cell marker (ACTA2), and macrophage markers (CD68 and LGALS3). Cholesterol loading of VSMCs significantly increased macrophage‐related gene expression and decreased VSMC‐related gene expression (Fig. 1C). In parallel, the absence of ACTA2 immunostaining (Fig. 1D) and the increase in CD68 immunostaining (Fig. 1E) confirmed that VSMCs converted to a macrophage‐like cell after cholesterol loading.

### CTRP9 alleviates cholesterol loading‐induced VSMC pro‐inflammatory cytokine secretion and suppresses THP1 cell adhesion to VSMCs

**Figure 2:**
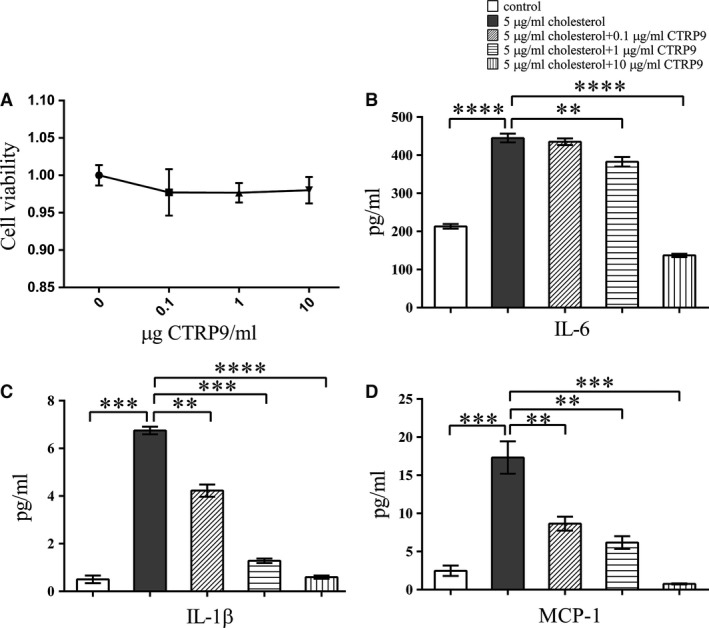
CTRP9 treatment alleviates cholesterol loading‐induced VSMC pro‐inflammatory factor secretion (**A**) Cell viability of VSMCs, measured using CCK8, after incubation with CTRP9 for 72 hrs. (**B**‐**D**) VSMCs were treated for 72 hrs with different concentrations of CTRP9 (0–10 μg/ml) in the presence of 5 μg/ml cholesterol or were left untreated (control) before measurement of (**B**) IL‐6 secretion, (**C**) IL‐1β secretion and (**D**) MCP‐1 secretion. Values represent the means ± S.D. of triplicate reactions and are representative of three independent experiments. **P < 0.01, ***P < 0.001, ****P < 0.0001.

Local pro‐inflammatory factors in plaques play important roles in atheroma progression. To clarify the potential role of VSMC‐derived macrophage‐like cells in atherosclerosis, we measured the levels of pro‐inflammatory cytokines in VSMC supernatants. VSMCs were either left untreated or were treated with 5 μg/ml cholesterol in the presence of different concentrations of CTRP9. Cholesterol loading of VSMCs clearly increased the levels of secreted IL‐6 (Fig. 2B), IL‐1β (Fig. 2C) and MCP‐1 (Fig. 2D). Treatment of VSMCS with up to 10 μg/ml CTRP9 did not affect cell viability (Fig. 2A); however, CTRP9 pre‐treatment suppressed cholesterol‐induced pro‐inflammatory cytokine secretion in a dose‐dependent manner (Fig. 2B–D).

**Figure 3:**
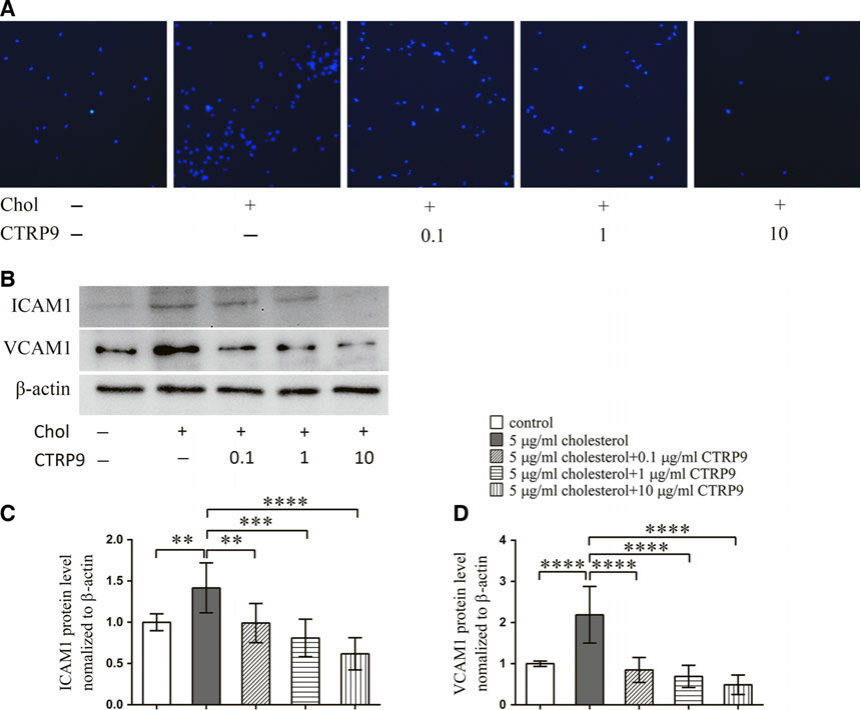
CTRP9 treatment alleviates cholesterol loading‐induced THP1 cell adhesion on VSMCs VSMCs were treated for 72 hrs with different concentrations of CTRP9 (0–10 μg/ml) in the presence of 5 μg/ml cholesterol or were left untreated. (**A**) Representative images of THP1 cell adhesion to the differently treated VSMCs (50×). (**B**) Representative Western blot to examine expression of the cell adhesion molecules ICAM‐1 and VCAM‐1 in the differently treated VSMCs. (**C**,** D**) Quantification of Western blot images. Values represent the means ± S.D. of triplicate reactions and are representative of three independent experiments. **P < 0.01, ***P < 0.001, ****P < 0.0001.

To understand the effect of cholesterol on the ability of VSMCs to recruit monocytes, we used a cell adhesion assay employing CELLTRACE Violet‐labelled THP1 cells and VSMCs. THP1 monocyte adhesion to cholesterol‐treated VSMCs was significantly greater than adhesion to untreated VSMCs. CTRP9 pre‐treatment reduced this monocyte adhesion in a dose‐dependent manner (Fig. 3A). To further explore the mechanism by which cholesterol‐loaded VSMCs recruited monocytes, we measured VCAM‐1 and ICAM‐1 expression in VSMCs by Western blotting. In VSMCs, cholesterol loading significantly increased the expression of VCAM‐1 and ICAM‐1, whereas CTRP9 pre‐treatment completely prevented this effect (Fig. 3B–D).

### CTRP9 inhibits VSMC cholesterol uptake and promotes the expression of cholesterol efflux‐related molecules

**Figure 4:**
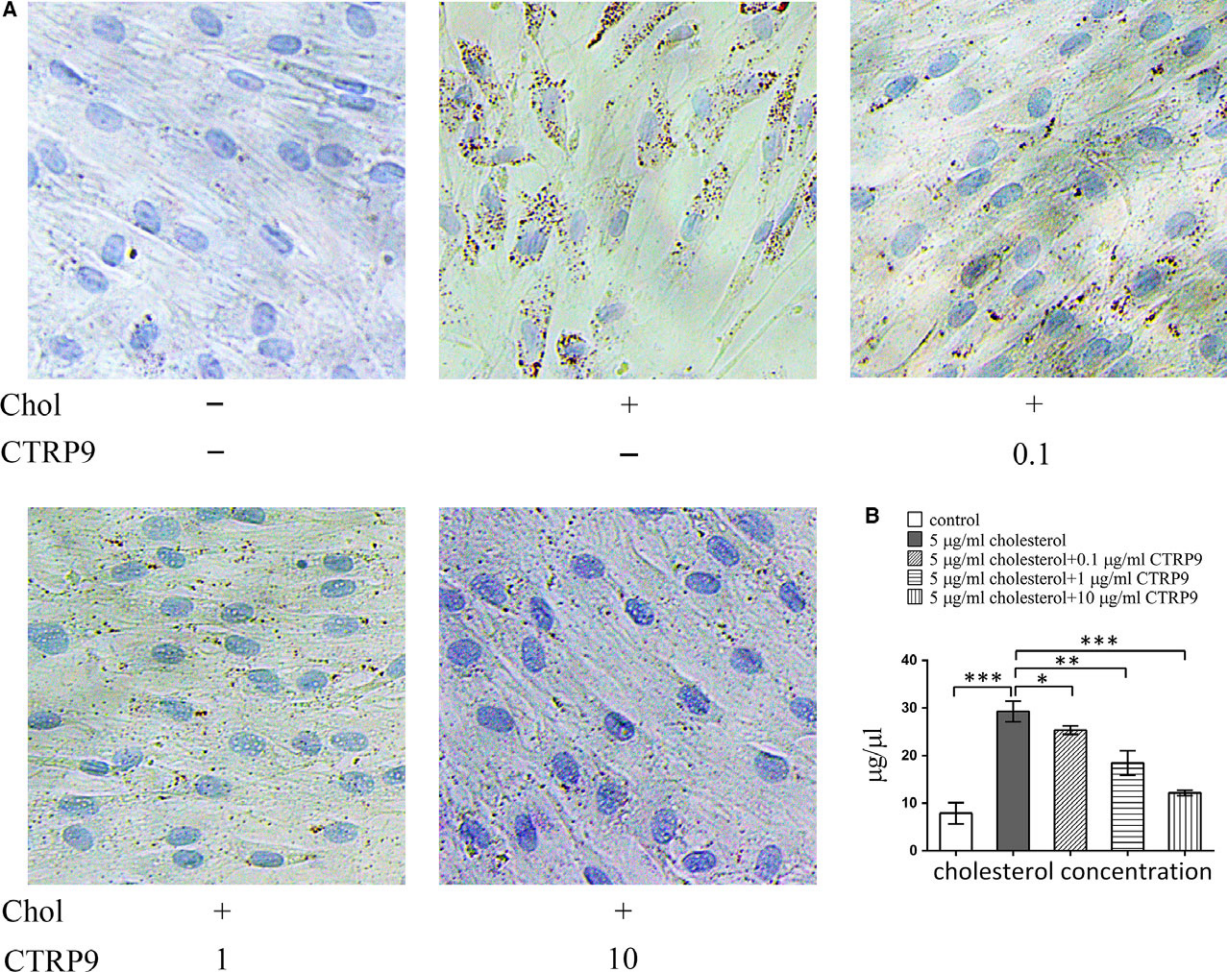
CTRP9 decreases cholesterol loading in VSMCs VSMCs were treated for 72 hrs with different concentrations of CTRP9 (0–10 μg/ml) in the presence of 5 μg/ml cholesterol or were left untreated (control). (**A**) Representative images from Oil Red O and Harris’ hematoxylin staining (200×). (**B**) Quantification of cholesterol levels in the differently treated VSMCs. Values represent the mean ± S.D. and are representative of three independent experiments. *P < 0.05, **P < 0.01, ***P < 0.001.

The appearance of Oil‐Red‐O‐stained lipid droplets in cholesterol‐treated VSMCs was inhibited by CTRP9 pre‐treatment in a dose‐dependent manner and was almost completely undetectable after pre‐treatment with 10 μg/ml CTRP9 (Fig. 4A). The level of VSMC cholesterol in cholesterol‐loaded VSMCs was also reduced in a dose‐dependent manner by CTRP9 pre‐treatment and was almost completely reversed by pre‐treatment with 10 μg/ml CTRP9 (Fig. 4B).

**Figure 5:**
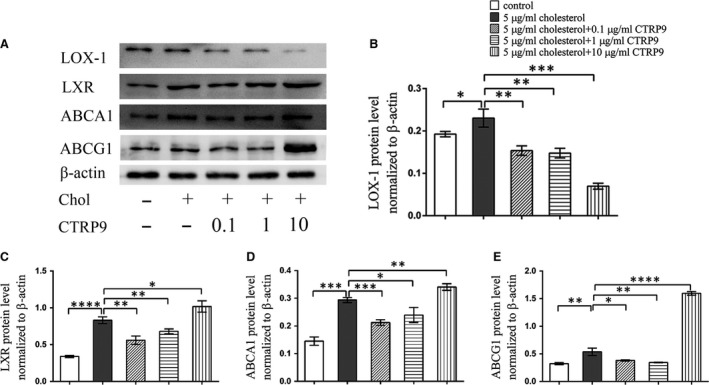
CTRP9 regulates cholesterol metabolism‐related proteins expression in VSMCs VSMCs were treated for 72 hrs with different concentrations of CTRP9 (0–10 μg/ml) in the presence of 5 μg/ml cholesterol or were left untreated (control). (**A**) Representative Western blot of differently treated VSMCs to examine LOX‐1, LXR, ABCA1 and ABCG1 expression. (**B**–**E**) Quantification of Western blot images. Values represent the mean ± S.D. and are representative of three independent experiments. *P < 0.05, **P < 0.01, ***P < 0.001, ****P < 0.0001.

Western blot analysis revealed that cholesterol loading upregulated the expression of LOX‐1, while 0.1 μg/ml CTRP9 prevented this upregulation; higher concentrations of CTRP9 reduced LOX‐1 levels even further (Fig. 5A and B). Cholesterol loading of VSMCs increased the expression of the cholesterol efflux proteins LXR, ABCG1 and ABCA1. Interestingly, low concentrations of CTRP9 decreased LXR, ABCA1 and ABCG1 expression in VSMCs relative to cholesterol‐only treated VSMCs, whereas higher concentrations of CTRP9 increased the expression of these proteins beyond the cholesterol‐only group (Fig. 5A, C–E).

### CTRP9 suppresses the cholesterol loading effect on VSMC phenotypic trans‐differentiation

**Figure 6:**
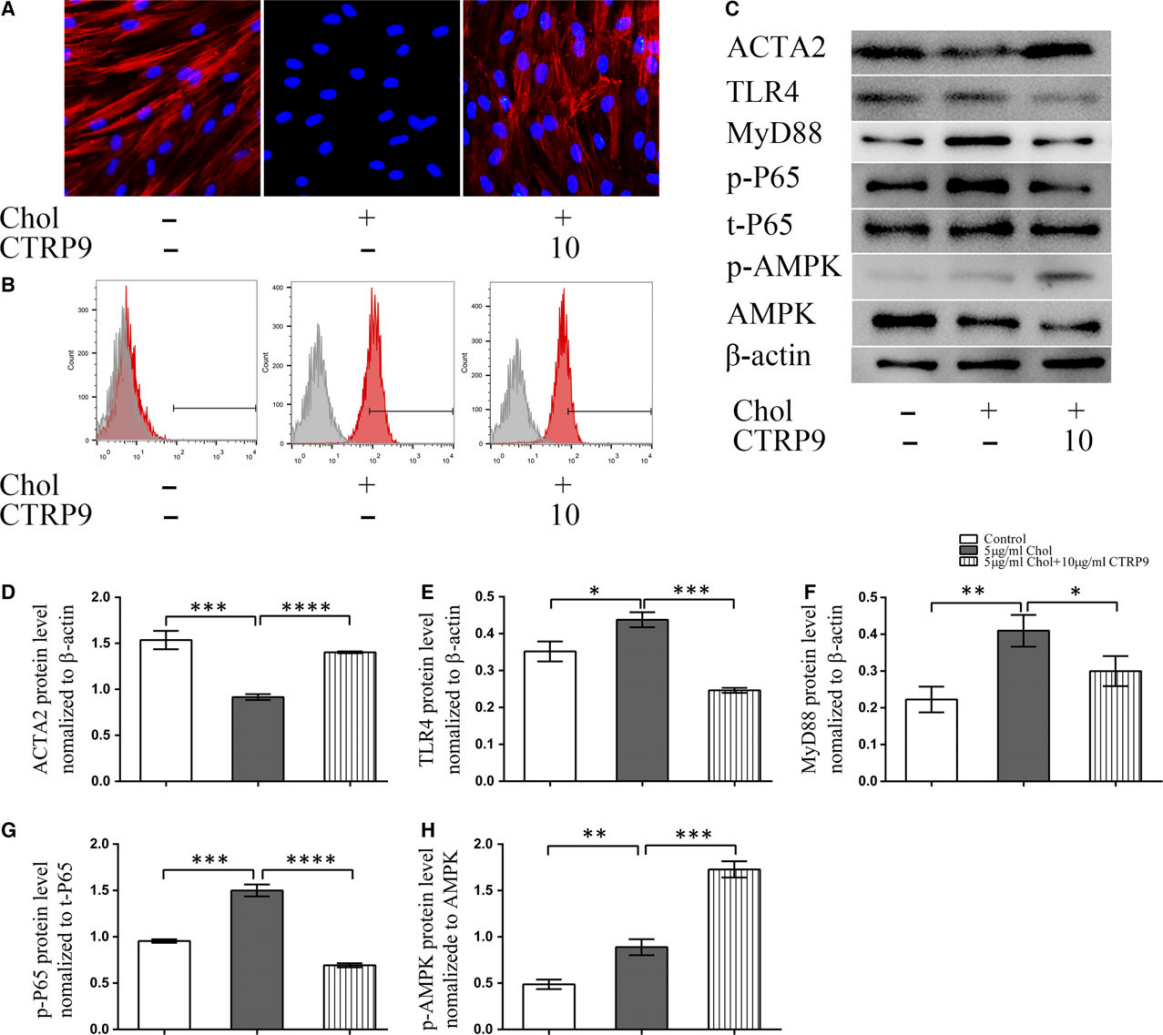
CTRP9 inhibits the VSMC switch to macrophage‐like cells, activates the AMPK pathway and suppresses cholesterol‐induced NF‐kB pathway activation VSMCs were left untreated (control) or were treated with 5 μg/ml cholesterol in the absence or presence of 10 μg/ml CTRP9 for 72 hrs. (**A**) Representative fluorescence images showing ACTA2 expression levels in VSMCs under the different treatment conditions. (Red: ACTA2 Blue: DAPI) (200×) (**B**) Representative flow cytometry analysis of CD68‐positive cells. (**C**) Representative Western blot to examine ACTA2, TLR4, MYD88, phospho‐p65 (p‐p65), total p65 (t‐p65), phospho‐AMPK (p‐AMPK) and total AMPK (AMPK) in VSMCs from each treatment. (**C**–**H**) Quantification of Western blot images. Values represent the means ± S.D. of triplicate reactions and are representative of three independent experiments. *P < 0.05, **P < 0.01, ***P < 0.001, ****P < 0.0001.

After a 72‐ hrs incubation with cholesterol, ACTA2 immunostaining was absent in VSMCs (Fig. 6A), and pre‐treatment with 10 μg/ml CTRP9 prevented this loss (Fig. 6A). To further characterize this cholesterol‐induced VSMC phenotypic switch, the expression of CD68‐positive VSMCs was examined using flow cytometry. Cholesterol treatment for 72 hrs induced 72.1% of the VSMCs to adopt a macrophage‐like phenotype, whereas only 23.0% of cells adopted this phenotype following pre‐incubation with 10 μg/ml CTRP9 and cholesterol (Fig. 6B).

### Cholesterol loading modulates the NF‐κB and AMP‐activated protein kinase (AMPK) pathways in VSMCs

To further understand the mechanism of cholesterol loading‐induced VSMC cell dysfunction, we measured the protein expression of VSMC markers, AMPK phosphorylation and NF‐κB pathway‐related proteins using Western blotting. Cholesterol loading clearly upregulated NF‐κB‐related protein expression in VSMCs, and this was accompanied by a small increase in AMPK phosphorylation, as well as the significant decrease previously described in ACTA2 expression (Fig. 6C–H). Compared with cholesterol‐loaded VSMCs, the expression levels of TLR4, MYD88 and the level of P65 phosphorylation were all reduced by pre‐incubation with 10 μg/ml CTRP9 (Fig. 6C–G); in contrast, ACTA2 levels and the level of p‐AMPK phosphorylation were both increased by CTRP9 pre‐treatment (Fig. 6C and H).

### Knockdown of AMPK α1 abolished CTRP9′s protective effect on VSMCs

**Figure 7:**
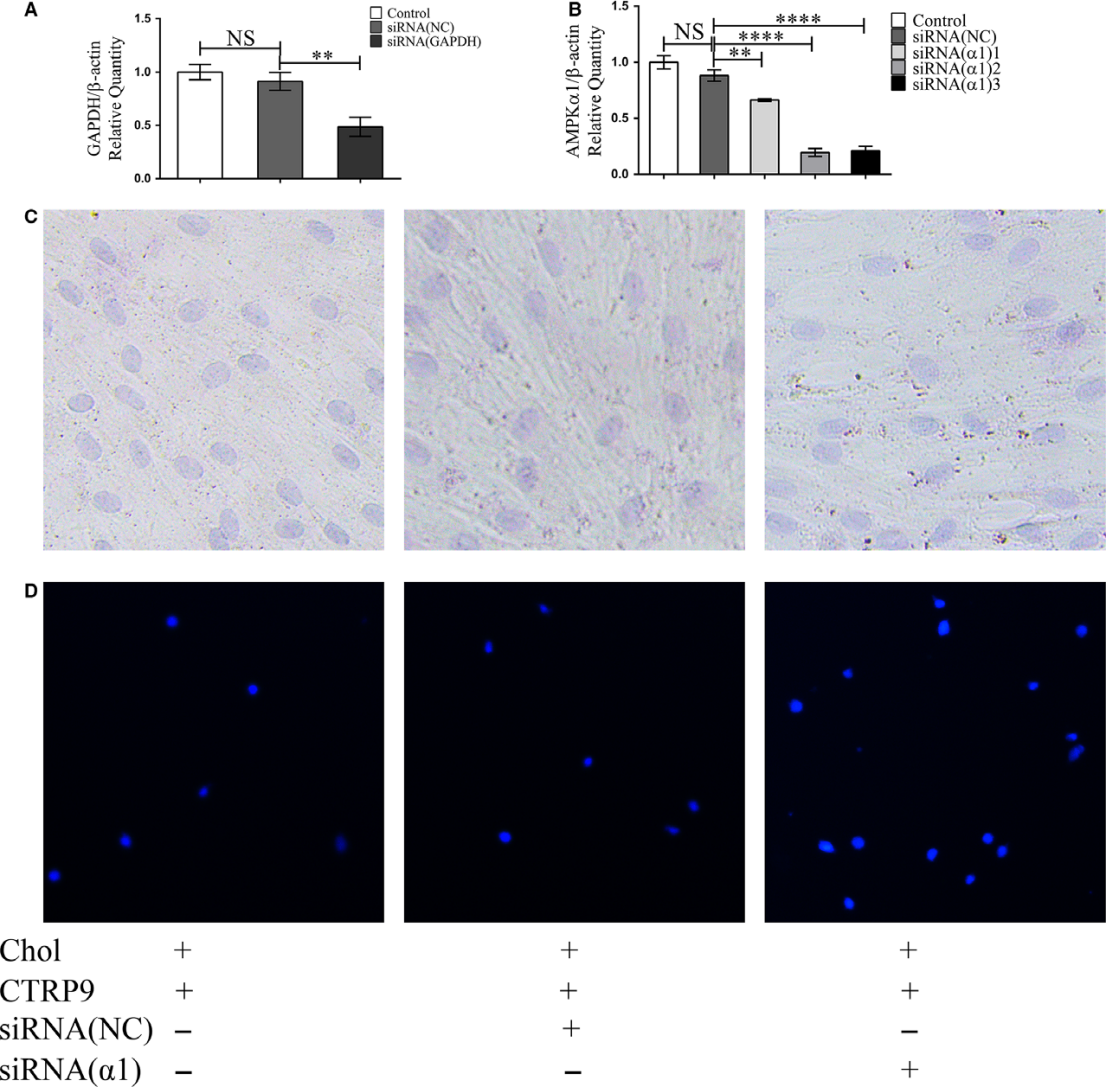
Knockdown of AMPKα1 abolishes CTRP9 counteractive cholesterol loading in VSMCs effect and increased THP1 cell adhesion on VSMC. VSMCs were transfected with an siRNA targeting AMPα1 (siRNAα1), a control siRNA (NC), or were not transfected (‐). Cells were then treated with 5 μg/ml cholesterol for 72 hrs in the presence of CTRP9 (10 μg/ml). (**A**) mRNA expression of GAPDH measured by qRT‐PCR in VSMC cells after transfection with siRNA(NC) or siRNA(GAPDH) for 36 hrs. (**B**) mRNA expression of AMPKα1 measured by qRT‐PCR in VSMC cells after transfection with siRNA(NC) or siRNA(α1) for 36 hrs. (**C**) Representative images from Oil Red O staining and Harris’ hematoxylin staining of cells (200×). (**D**) Representative images of THP1 cell adhesion to the differently treated VSMCs (50×). qRT‐PCR (**B**) data are shown as mean ± S.D. of triplicates and are representative of three independent experiments. **P < 0.01.

**Figure 8:**
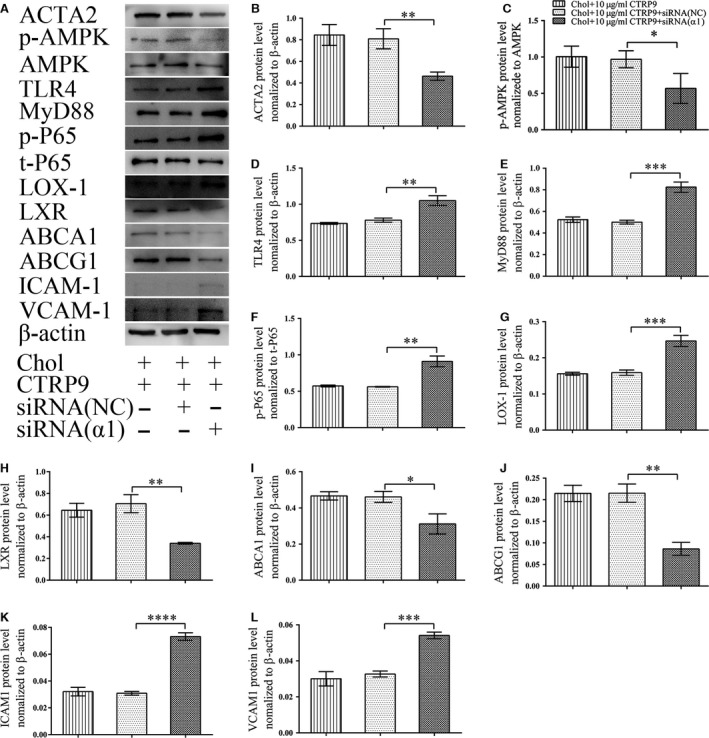
Knockdown of AMPKα1 abolishes CTRP9 protective effects in VSMCs VSMC cells were transfected with an siRNA targeting AMPα1 (siRNAα1), a control siRNA (NC), or were not transfected (‐). Cells were then treated with 5 μg/ml cholesterol in the presence of CTRP9 (10 μg/ml) for 72 hrs. (**A**) Representative Western blot of ACTA2, phospho‐AMPK (p‐AMPK), total AMPK (AMPK), TLR4, MyD88, phospho‐p65 (p‐p65), total p65 (t‐p65), LOX‐1, LXR, ABCA1, ABCG1, ICAM‐1 and VCAM‐1 in VSMCs from each treatment. (**B**–**L**) Quantification of Western blot images. Values represent the means ± S.D. of triplicate reactions and are representative of three independent experiments. *P < 0.05, **P < 0.01, ***P < 0.001, ****P < 0.0001.

To further explore the role of AMPK in this CTRP9 protective effect, lipid deposition and monocyte adhesion to VSMCs were measured after knockdown of AMPK α1 by siRNA. AMPK α1 knockdown significantly decreased the level of AMPK α1 (Fig. 7A and B) expression and prevented the CTRP9‐induced protection of cholesterol‐loaded VSMCs as evidenced by the accumulation of Oil‐Red‐O‐stained lipid droplets and the augmentation of monocyte adhesion (Fig. 7C). The effects of AMPK α1 knockdown on AMPK phosphorylation, the NF‐ κB pathway, cholesterol metabolism‐related proteins and monocyte adhesion‐related proteins were examined by Western blotting. Along with decreased ACTA2 expression, knockdown of AMPKα1 decreased AMPK phosphorylation and upregulated TLR4, MYD88 expression and p‐P65 levels in VSMCs. Following AMPK knockdown in VSMCs, the expression of LOX‐1, NF‐kB and monocyte adhesion‐related proteins increased, whereas the expression of LXR, ABCA1 and ABCG1 decreased (Fig. 8).

**Figure 9:**
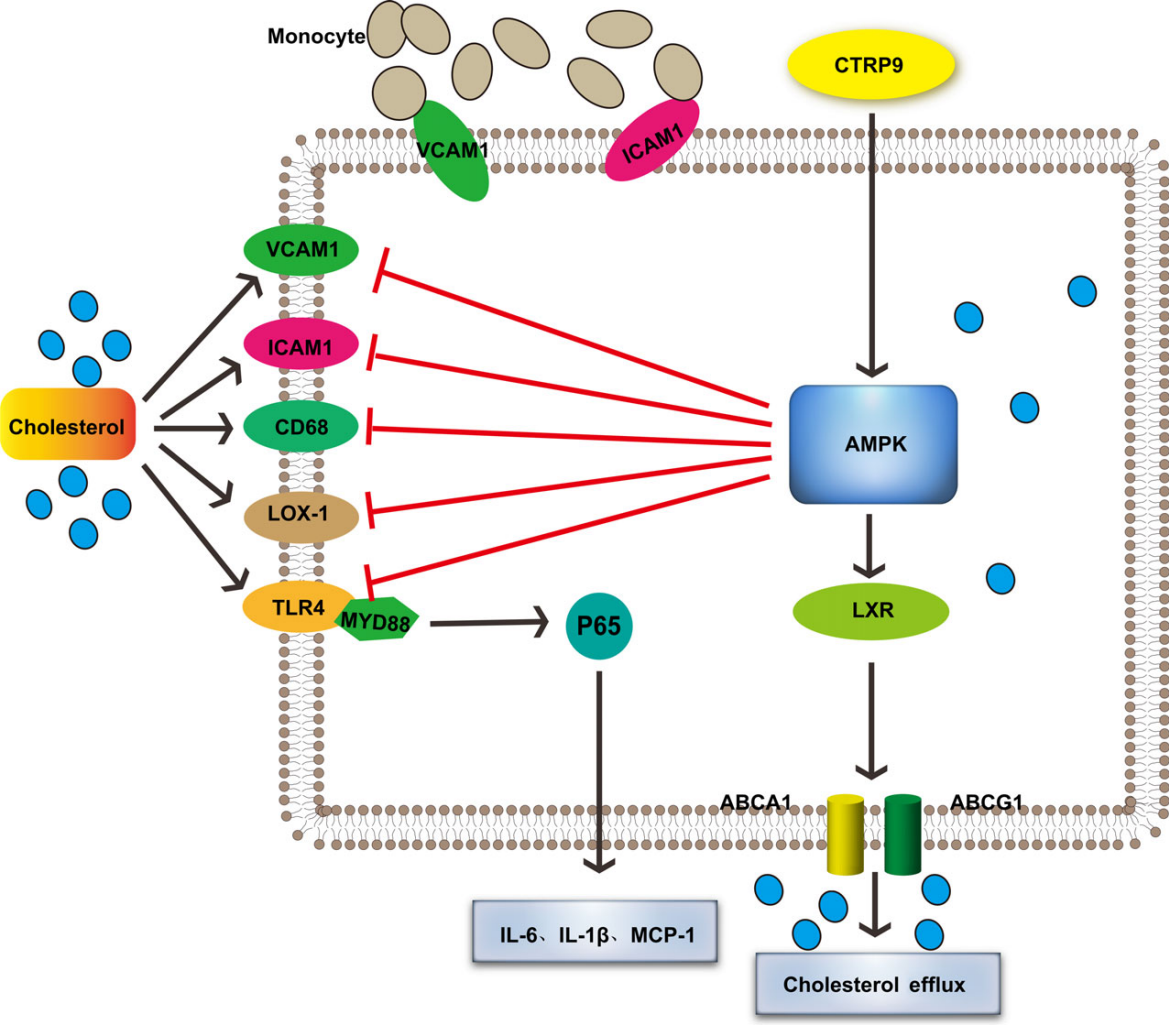
A model showing the possible mechanism of CTRP9‐mediated protection of VSMCs against cholesterol‐induced injury through AMPK activation.

## Discussion

The current study demonstrates, for the first time, that CTRP9 can act as a powerful protector to prevent cholesterol‐induced damage in VSMCs. CTRP9 decreased the total intracellular cholesterol content indicating that CTRP9 prevented VSMC‐derived macrophage‐like foam cell formation and subsequent changes in biological function.

The existence of smooth muscle foam cells in human atherosclerosis has been well documented 29; recent studies have demonstrated that cholesterol loading downregulates VSMC contractile proteins, but upregulates macrophage markers, which indicates a switch of VSMCs to macrophage‐like foam cells 5, 8, 30. In addition to this VSMC phenotypic change, this study demonstrated that cholesterol loading induces the secretion of pro‐inflammatory cytokines such as IL‐6, IL‐1β and MCP‐1, and also induces monocyte adhesion to VSMCs. Inflammation plays a central role in all phases of atherogenesis, from the initial recruitment of innate and acquired immune cells to infiltrate the arterial wall, to later athero‐thrombotic events 31, 32. The hypothesis that reducing pro‐inflammatory factors reduced both atherosclerotic plaque development and plaque destabilization has been confirmed 33, 34. With regard to the pro‐inflammatory factors secreted by VSMCs after cholesterol loading, it is possible that VSMC‐derived macrophage‐like cells might play an important role in accelerating plaque development and if so inhibiting the dysfunction in VSMCs caused by cholesterol loading might be a meaningful way to inhibit atherosclerosis. Our *in vitro* results demonstrated that CTRP9 decreases cholesterol loading‐induced pro‐inflammatory cytokine secretion by VSMCs, suggesting that CTRP9 may indeed be effective in suppressing atherosclerosis.

Recruitment of monocytes into the wall of large arteries accelerates atherosclerotic progression 35. Cholesterol loading upregulated ICAM‐1 and VCAM‐1 expression and at the same time promoted monocyte adhesion to VSMCs. CTRP9 pre‐treatment prevented both of these cholesterol‐induced effects, thus suggesting that CTRP9 suppresses monocyte recruitment to VSMCs through the inhibition of ICAM‐1 and VCAM‐1 expression.

Vengrenyuk *et al*. demonstrated that promoting cholesterol efflux facilitates macrophage‐like VSMCs to regain VSMC markers and *in vivo* this may contribute to the formation of VSMC‐containing fibrous caps 8. Thus, inhibiting cholesterol loading while promoting cholesterol efflux will protect VSMCs from cholesterol injury and provide a benefit to plaque stability. Numerous genes play roles in cholesterol uptake and efflux in cells. LOX‐1 is a receptor specific for ox‐LDL, upregulation of which promotes lipid loading; it therefore plays an important role in atherogenesis 36. TLR4 expressed by SMC contributes to foam cell formation in early atherogenesis 37. The nuclear receptor LXR plays an important role in both lipid metabolism and inflammation 38. The transporters ABCA1 and ABCG1 contribute to reverse cholesterol transport and are important in inhibiting plaque formation 39, 40. Interestingly, we found that cholesterol loading induced the upregulation of proteins involved in cholesterol uptake and reverse cholesterol transport. The levels of LOX‐1, TLR4, LXR, ABCA1 and ABCG1 were clearly increased in the VSMC macrophage‐like state. CTRP9 significantly decreased LOX‐1 and TLR4 expression in VSMCs, whereas high concentrations of CTRP9 further increased LXR, ABCA1 and ABCG1 expression. However, Oil‐Red‐O‐positive lipid drops were significantly decreased at low concentrations of CTRP9 and quantification of intracellular cholesterol confirmed that CTRP9 also effectively inhibits cholesterol loading in VSMCs. As we did not treat VSMCs with apolipoprotein A‐I (ApoA‐I) to promote intracellular cholesterol efflux, the decreased cholesterol levels in cholesterol‐loaded VSMCs treated with CTRP9 likely occur as a result of the inhibition of cholesterol intake through inhibition of Lox‐1 and TLR4 expression.

The transcription factor NF‐kB is associated with activation of inflammatory pathways and the onset of atherosclerosis 41. In fact, the expression of VCAM‐1 and ICAM‐1 in atherosclerotic lesions is principally controlled by NF‐kB 42, 43. Our results showed that the NF‐kB pathway participates in cholesterol‐induced VSMC damage. ICAM‐1 and VCAM‐1 expression were both increased in VSMCs following cholesterol loading. CTRP9 pre‐treatment significantly decreased the per cent of CD68‐positive VSMCs and prevented downregulation of ACTA2 after cholesterol loading, thereby demonstrating protection of the smooth muscle phenotype and inhibition of the macrophage phenotype.

AMPK, an energy sensor, is an important kinase that can be activated by various cellular stresses 44. Previous studies have shown that overexpression of CTRP9 enhances AMPK activation, whereas knockout of CTRP9 reduced AMPK activation 11, 12, 18, 22. Consistent with this, CTRP9 pre‐treatment of VSMCs significantly enhanced the degree of phosphorylation of AMPK in cholesterol‐loaded cells. Once activated, AMPK phosphorylates and regulates several downstream proteins including LOX1 45, LXR 46, ABCA1 46 and ABCG1 47. Our results demonstrated that CTRP9 increased LXR, ABCA1 and ABCG1 expression in VSMCs, while decreasing LOX1 levels. The slight increase in the level of phosphorylated AMPK in cholesterol‐loaded VSMCs may be associated with the increase in LXR, ABCA1 and ABCG1, which is also seen in cholesterol‐loaded VSMCs. It has been reported that AMPK 48, 49 and LXR 50, 51 both inhibit NF‐kB activation. While CTRP9 pre‐treatment inhibited cholesterol‐induced TLR4, MYD88 and p‐P65 expression in VSMCs, this was accompanied by decreased expression of ICAM1 and VCAM1. Nevertheless, knockdown of AMPKα1 in VSMCs abolished CTRP9‐induced ACTA2 upregulation, AMPK phosphorylation, LOX1 downregulation, downregulation of NF‐kB‐related proteins and upregulation of LXR, ABCA1 and ABCG1. These results indicate that AMPK activation is crucial in the pathway by which CTRP9 protects VSMCs from cholesterol‐induced damage. A limitation in the present study was that the results were not examined *in vivo*, which therefore needs further investigation.

In conclusion, we present data suggesting that cholesterol loading of VSMCs augments the expression of inflammatory factors and monocyte adhesion, which together may increase plaque lipid burden. CTRP9 inhibits uptake of cholesterol by VSMCs and therefore counteracts the cholesterol loading‐induced VSMC dysfunction. Thus, CTRP9 appears to be a promising drug to treat atherosclerosis through regulation of VSMC function.

## Conflict of interests

The authors confirm that there are no conflict of interests.

## Author's Contribution

Q.L., H.Z., J.L., W.L. and M.S. performed experiments; R.Z. and S.C. performed statistical analyses. B.Y. and W.D. supervised the experiments and reviewed the manuscript. Q.L. and J.H. designed the project and wrote the manuscript.
